# Comparison of the procedure time differences between hybrid endoscopic submucosal dissection and conventional endoscopic submucosal dissection in patients with early gastric neoplasms: a study protocol for a multi-center randomized controlled trial (Hybrid-G trial)

**DOI:** 10.1186/s13063-022-06099-x

**Published:** 2022-02-21

**Authors:** Mitsuru Esaki, Eikichi Ihara, Hiroyuki Fujii, Yorinobu Sumida, Kazuhiro Haraguchi, Shunsuke Takahashi, Tsutomu Iwasa, Kayoko Nakano, Masafumi Wada, Shinichi Somada, Yosuke Minoda, Haruei Ogino, Koshiro Tagawa, Yoshihiro Ogawa

**Affiliations:** 1 Department of Medicine and Bioregulatory Science, Graduate School of Medical Sciences, Kyushu University, Fukuoka, 8128582 Japan; 2 Department of Gastroenterology and Metabolism, Graduate School of Medical Sciences, Kyushu University, 3-1-1 Maidashi, Higashi-ku, Fukuoka, 8128582 Japan; 3 Department of Gastroenterology and Hepatology, National Hospital Organization Fukuokahigashi Medical Center, Koga, 8113195 Japan; 4 Department of Gastroenterology, Clinical Research Institute, National Hospital Organization Kyushu Medical Center, Fukuoka, 8108563 Japan; 5 Department of Gastroenterology, Hara-sanshin Hospital, Fukuoka, 8120033 Japan; 6 Department of Gastroenterology, Fukuoka City Hospital, Fukuoka, 8111394 Japan; 7 Department of Gastroenterology, Fukuokaken Saiseikai Futsukaichi Hospital, Fukuoka, 8118516 Japan; 8 Department of Gastroenterology, Fukuoka Central Hospital, Fukuoka, 8100022 Japan; 9 Department of General Internal Medicine, Saiseikai Yahata General Hospital, Fukuoka, 8050050 Japan; 10 Department of Gastroenterology, National Hospital Organization Beppu Medical Center, Beppu, Oita 8740011 Japan; 11 Center for Clinical and Translational Research, Kyushu University Hospital, Fukuoka, 8128582 Japan

**Keywords:** Hybrid endoscopic submucosal dissection, Conventional endoscopic submucosal dissection, Early gastric neoplasms

## Abstract

**Background:**

Endoscopic submucosal dissection (ESD) is widely accepted as a local treatment for gastrointestinal tract tumors. As a simplified endoscopic procedure, hybrid ESD (H-ESD) has been performed for colorectal neoplasms in recent times. However, whether H-ESD is superior to conventional ESD (C-ESD) for patients with early gastric neoplasms (EGN) remains unclear. In this trial, we will compare the treatment outcomes of H-ESD and C-ESD. We hypothesize that the procedure time for H-ESD is shorter than that for C-ESD.

**Methods:**

This is an investigator-initiated, multi-center, prospective, randomized, open-label, parallel-group trial to be conducted beginning in August 2020 at nine institutions in Japan. We will determine if H-ESD is superior to C-ESD in terms of procedure time in patients with EGN diagnosed as macroscopically intramucosal (T1a) differentiated carcinoma ≤ 20 mm in diameter without ulcerative findings according to current Japanese gastric cancer treatment guidelines. A total of 82 patients will be recruited and randomly assigned to either the C-ESD or the H-ESD group. The primary outcome is ESD procedure time. Secondary outcomes include mucosal incision, time and speed of submucosal dissection, en bloc resection, complete resection, curability, adverse events related to the ESD procedure, extent of dissection before snaring, volume of injection solution, number and time of hemostasis, thickness of the submucosal layer in the resected specimen, and handover to another operator. The stated sample size was determined based on the primary outcome. According to a previous report comparing the procedure times of C-ESD and H-ESD, we hypothesized that H-ESD would provide a 0.2 reduction in logarithmically concerted procedure time (−37%). We estimated that a total of 82 participants were needed to reach a power of 80% for a *t*-test with a significance level of 0.05 and considering a 10% dropout.

**Discussion:**

This trial will provide high-quality data on the benefits and risks of H-ESD for EGN patients. The results of this study could lead to improved outcomes in patients with EGN undergoing ESD. The results will be presented at national and international meetings and published in peer-reviewed journals.

**Trial registration:**

UMIN-CTR UMIN000041244. Registered on July 29, 2020.

## Background

Endoscopic resection (ER) has become popular as a local treatment for early gastric neoplasms (EGN) without lymph node metastasis. ER is less invasive than surgery and preserves organ function, contributing to maintaining the patient’s quality of life after treatment [1]. Endoscopic mucosal resection (EMR) using an endoscopic steel snare, a simple technique that can be performed quickly, was first developed as an ER technique for EGN [2]. However, snaring for resections of lesions larger than 20 mm or ulcerated lesions presents a technical limitation in that lesions of this size approximately correspond to the diameter of the snare. EMR for such lesions results in a high rate of piecemeal resections, associated with a high local recurrence rate and difficulty in accurate histological assessment [3].

Later, endoscopic submucosal dissection (ESD) using an endoscopic steel knife (endo-knife) was developed for circumferential incision and subsequent submucosal dissection from the proper muscle layer [4]. ESD allows for en bloc resections, even for lesions larger than 20 mm and/or ulcerated lesions, and accurate histological assessments.

According to the current Japanese gastric cancer treatment guidelines, “macroscopically intramucosal (T1a) differentiated carcinoma ≤ 20 mm in diameter without ulcerative findings” is defined as an “absolute indication of EMR or ESD” [5, 6], while lesions larger than 20 mm and/or with ulcerative findings are indicated as an “absolute indication of ESD” or an “expanded indication of ESD” based on the lesion size and presence or absence of ulcerative findings. Previous meta-analyses have suggested that the en bloc resection rate of EMR is significantly lower than that of ESD when gastric lesions exceed 10 mm in size [7–9]; however, previous reports have also suggested that ESD for EGN is more time-consuming and carries a higher risk of perioperative complications due to the requirement of specialized skills needed to perform the procedure [9]. Currently, in clinical practice, considering curability, rather than procedure time and/or risk of complications, ESD is likely to be selected in most cases, even when the gastric lesion meets the absolute indications for EMR or ESD [10].

In this situation, a modified endoscopic procedure called hybrid ESD (H-ESD) has been developed by fusing ESD with EMR. This procedure involves making a circumferential mucosal incision with a subsequent partial submucosal dissection as part of the ESD procedure, followed by snaring, as with EMR. H-ESD is a simplified procedure involving planned snaring during submucosal dissection, and it shows high curability because of the ESD portion of the procedure and a shorter procedural time due to the EMR. Indeed, H-ESD has already been used for colorectal neoplasms, and favorable outcomes with significantly shorter procedure times than with conventional ESD (C-ESD) have been reported [11].

However, the superiority of H-ESD over C-ESD for EGN is still controversial. One retrospective study has shown a significant reduction in procedure time and favorable treatment outcomes with H-ESD, compared with C-ESD [12]. However, another retrospective study failed to show a significantly shorter procedure time for H-ESD [13]. Therefore, it remains unclear whether H-ESD is superior to C-ESD for the treatment of EGN. To address this issue, we aim to conduct a multi-center, randomized controlled trial comparing the clinical outcomes of H-ESD and C-ESD for EGN.

## Methods

### Objective

This study aims to clarify the clinical position of H-ESD for the standard treatment of EGN and compare the treatment efficacies of H-ESD and C-ESD for EGN.

### Trial design

**Fig. 1:**
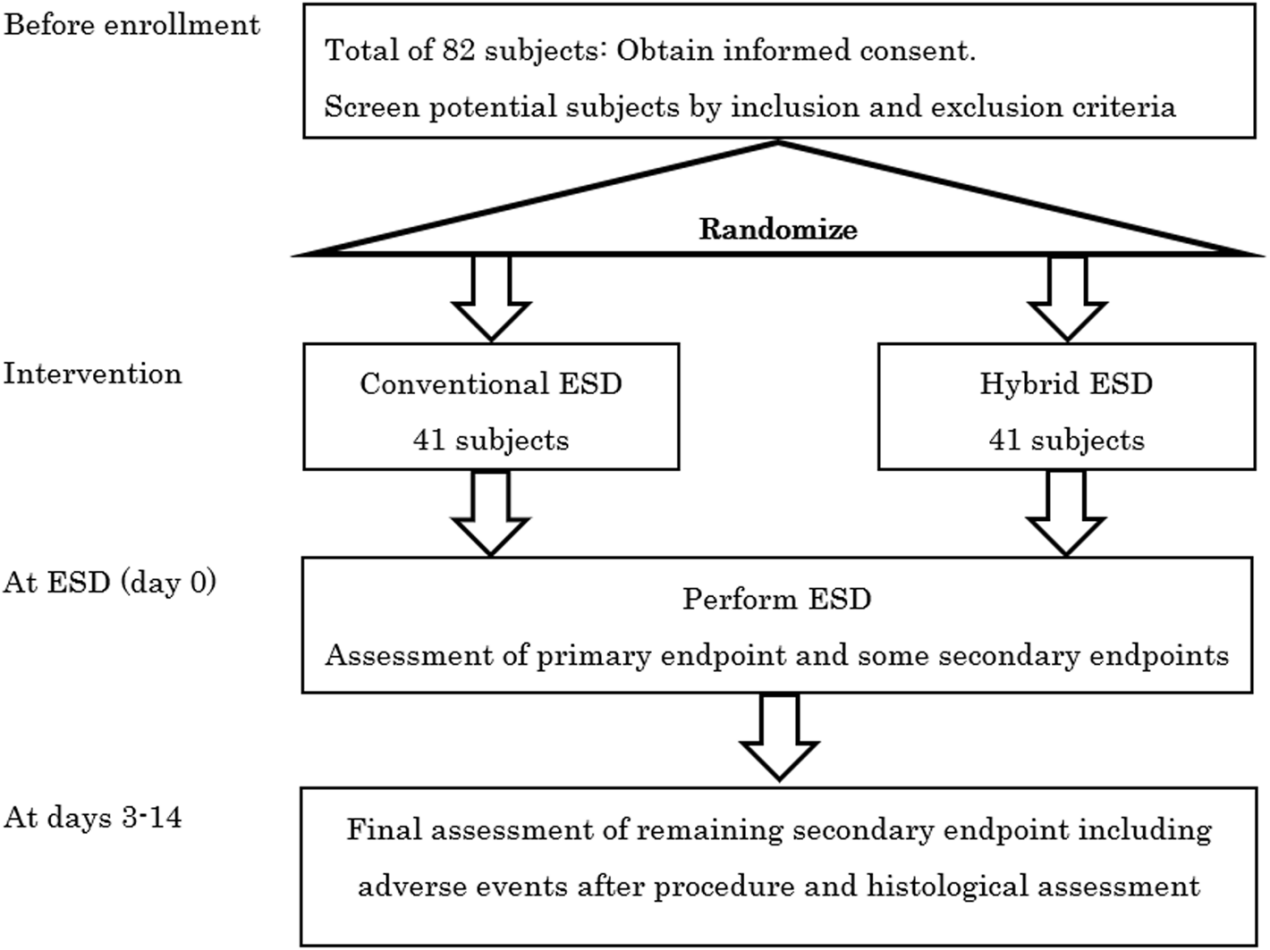
Schematic of the study design. ESD, endoscopic submucosal dissection

**Fig. 2:**
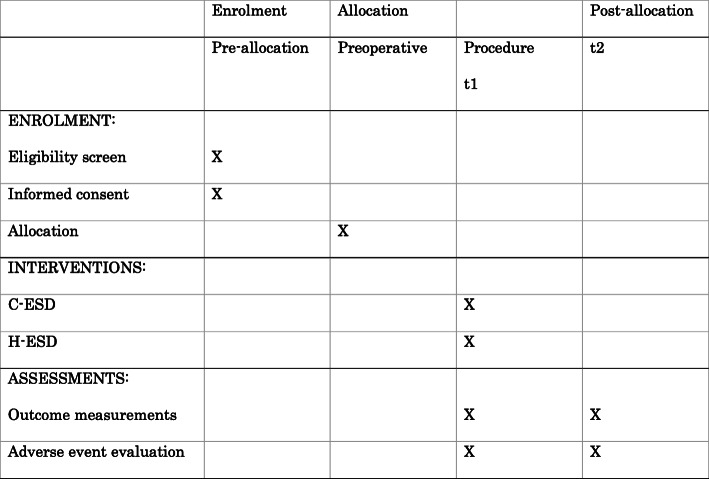
SPIRIT flow diagram. H-ESD, hybrid endoscopic submucosal dissection; C-ESD, conventional endoscopic submucosal dissection

We will conduct a prospective, parallel, randomized, open-label, superiority trial as per the recommendations found in the Standard Protocol Items: Recommendations for Interventional Trials (SPIRIT) checklist [14]. We will report the results in accordance with the Consolidated Standards of Reporting Trials (CONSORT) statement. A flow chart of this study design is shown in Fig. 1. A total of 82 patients with EGN will be recruited and randomly assigned (1:1 ratio) to undergo either H-ESD or C-ESD. The SPIRIT flow diagram is shown in Fig. 2.

### Settings

This multi-center trial will include the following nine hospitals in Japan that have a high endoscopic procedure volume: Kyushu University, National Hospital Organization Kyushu Medical Center, National Hospital Organization Fukuokahigashi Medical Center, Hara-sanshin Hospital, Fukuoka City Hospital, Fukuokaken Saiseikai Futsukaichi Hospital, Fukuoka Central Hospital, Saiseikai Yahata General Hospital, and National Hospital Organization Beppu Medical Center.

### Approvals

This trial has been approved by the Institutional Review Board (IRB) of Kyusyu University for clinical trials (IRB No. 20202005; July 28, 2020) and also by the IRBs of each participating institution. This trial has been registered at UMIN-CRT (ID: 000041244; July 29, 2020). The trial protocol (vol. 1.0; June 1, 2020) was designed in accordance with the SPIRIT guidelines. A SPIRIT checklist is provided in Additional file 1. Attending investigators will obtain informed consent from each patient who volunteers to participate in this study and who meets the eligibility criteria. If any protocol modifications occur, they will be communicated to all study personnel on time.

### Population

#### Inclusion criteria

The inclusion criteria are as follows: (i) age ≥20 years of any sex; (ii) presence of EGN and capable of undergoing ESD as a local treatment; (iii) lesions diagnosed by endoscopic biopsy as gastric adenomas or adenocarcinomas; (iv) lesions endoscopically diagnosed as mucosal lesions ≤20 mm in diameter without ulceration ≤3 months prior to enrollment; (v) Eastern Cooperative Oncology Group Performance Status of 0–2; (vi) capable of listening to oral explanations and reading explanatory documents regarding doctors’ instructions as well as signing consent forms (including the informed consent that will be obtained before enrollment in the trial); and (vi) willingness to comply with all study procedures and be available for the duration of the study.

#### Exclusion criteria

Patients who meet at least one of the following exclusion criteria are considered ineligible for this trial: history of gastric surgery, are currently undergoing dialysis, or requiring continuous administration of heparin during the perioperative period. The concomitant use of other antithrombotic agents will be permitted during the study period. The Japanese guidelines for gastroenterological endoscopy for patients undergoing antithrombotic treatment will be adopted for the management of antithrombotic agents to the enrolled patients [15]. Those with contraindications for endoscopy, ileus, gastrointestinal perforation, or severe respiratory/cardiac disease are also excluded as are patients with metal allergies and those judged to be inappropriate for the study by the attending physician in this trial. Those who do not provide informed consent will also be excluded.

### Recruitment and consent

The patients will be screened by experienced endoscopists in each institution according to the inclusion and exclusion criteria. The endoscopists will assist patients in understanding the benefits and risks of the two treatment procedures, and written informed consent will be obtained from them prior to enrollment.

### Trial intervention

Enrolled patients will be randomized to undergo either H-ESD or C-ESD. Each patient with EGN will be treated using an assigned endoscopic procedure.

### Endoscopists and equipment

Each procedure will be performed at one of the nine institutions by one of the participating endoscopists; each endoscopist in this study has been licensed to practice medicine in Japan for at least 2 years and has performed ≥500 endoscopies. Endoscopists without experience in C-ESD or H-ESD will be required to undergo hands-on training using a pig model before becoming an operator in the study. Handover of the procedure to another operator will be allowed if experienced endoscopists consider the handover clinically desirable for reasons such as prolonged procedure time over 60 min, massive uncontrollable bleeding, or the occurrence of perforation during the procedure. Procedure handover will be done and recorded if the supervisor determines that it is necessary.

Upper gastrointestinal endoscopes (EG-450-RD5, Fujifilm, Tokyo, Japan; GIF-Q260J, Olympus, Tokyo, Japan) attached to a disposable hood (Elastic touch, Top, Tokyo Japan, or D-201-11804, Olympus) will be used in the ESD procedures. A Flush Knife (DK2620J, Fujifilm) or Dual Knife (KD-650, Olympus) will be used in the C-ESD procedure. A SOUTEN (ST1850-20, Kaneka Medix, Tokyo, Japan) will be used in the H-ESD procedure. The SOUTEN is a hybrid knife that combines a needle knife and a snare, where a needle tip is attached to the top of the snare. No regulation is required for the types of injection needles in this study. An electrosurgical unit (ICC200, VIO300, or VIO3, ERBE, Tubingen, Germany) will be used as a high-frequency generator. Sodium hyaluronate solution or sodium alginate solution will be used as a viscous solution for submucosal injections from the injection needle. Normal saline or glycerol will be used for submucosal injections using the endo-knives. Coagrasper (FD-410LR, Olympus), Coagrasper G (FD-412LR, Olympus), RAICHO (RC-1900, Kaneka Medix), RAICHO 2 (RC1550-2, Kaneka Medix), and Hemostat Y (H-S2518, Pentax, Japan) forceps will be used if bleeding cannot be controlled using the endo-knife.

### Endoscopic procedures

C-ESD and H-ESD procedures will be principally performed as en bloc resection. However, if en bloc resection is determined to be too difficult, preventive methods for local recurrence, such as piecemeal resection and additional coagulation using argon plasma coagulation or hot biopsy forceps, will be performed.

Both ESD procedures will be performed under sedation. C-ESD, described in detail elsewhere, consists of marking, injection, mucosal incision, and submucosal dissection [16, 17]. After observing the margin of the lesion, marking dots will be drawn 2–3 mm away from the margin. One of the viscous solutions will be injected into the submucosal layer using an injection needle to lift the target lesion. A circumferential mucosal incision will be made outside the marks. Subsequently, the submucosal layer will be dissected, and the target lesion will be retrieved. Marking, mucosal incision, and submucosal dissection will all be conducted using an endo-knife. There are no regulations for completing a circumferential mucosal incision before or during submucosal dissection in this study. The C-ESD procedure will be switched to traction-assisted ESD (TA-ESD) if the procedure time exceeds 60 min or if it is technically too difficult to ensure appropriate visualization and management of severe perforation or bleeding during C-ESD. However, TA-ESD is not considered a standard treatment because its superiority has not been shown in previous randomized controlled trials (RCTs) [18]. C-ESD can also be switched to H-ESD under the same conditions as those stated above; however, the selection of TA-ESD or H-ESD will depend on the discretion of the attending operator.

The H-ESD procedure will be performed, as described in detail elsewhere, using a SOUTEN high-frequency surgical knife [12, 19]. SOUTEN will be fixed with the snare closed, and only the distal tip will be exposed. Marking, injection, and mucosal incision will be performed in the same manner as described for C-ESD using the tip of the SOUTEN and an injection needle. After completing the circumferential incision, a partial submucosal dissection will be performed using the tip of the SOUTEN. Snaring of the lesion will be performed once the operator considers it possible to perform the resection in an en bloc manner. The open snare will be placed around the lesion, which will subsequently be resected by closing the snare and coagulation. H-ESD can also be switched to C-ESD if the operator considers snaring too difficult to perform. In such cases, the submucosal dissection will be performed until the specimen is retrieved.

Injection of the viscous material can be performed in both C-ESD and H-ESD using an injection needle, but it can also be done in C-ESD using an endo-knife. Hemostasis will be performed in both C-ESD and H-ESD using the tip of the devices if bleeding occurs or if the blood vessels are detected. If bleeding cannot be controlled using only an endo-knife, hemostatic forceps will be used.

Patients will be managed as after regular ESD, including being managed with intravenous nutrition and fasting. Patients will start to receive proton pump inhibitors, including potassium competitive acid blockers after ESD. Enteral diets will start within 2 or 3 days after ESD. If there are no serious adverse events (AEs), patients will be discharged approximately 1 week after ESD. If any AEs occur, investigators will immediately provide appropriate treatment and record it. The endoscopic treatment performed in this study is covered by the current medical insurance, and all costs associated with the study, including the treatment of AEs, will be charged and paid in accordance with the medical insurance system. There is no special compensation system for side effects caused by these treatments.

### Pathological assessment

Resected specimens will be fixed on a plastic plate and sliced at 2-mm intervals. Central pathological assessment will not be conducted for this trial. Pathologists at each institution will make the final pathological diagnoses based on the Japanese classifications of gastric carcinoma [20]. Researchers will obtain informed consent from patients for collecting biological samples for histopathological assessment. These samples will be stored strictly in a freezer in a locked laboratory for at least 5 years after the completion of the study and then properly disposed of in accordance with the Kyushu University Standard Operating Procedures for the Storage of Samples and Information Obtained from Human Subjects. Information will be stored in a password-protected computer in a locked laboratory. The person responsible for the management of personal information is YO Professor, Department of Medicine and Bioregulatory Science, Graduate School of Medical Sciences, Kyushu University. On the condition that this clinical trial is being conducted properly and confidentiality is maintained, samples and information of participants may be viewed by the monitoring, auditing, and ethical review committee personnel to whichever extent necessary.

### Outcome variables

#### Primary outcome

The primary outcome is the procedure time of each ESD among the full-analysis set (FAS). ESD procedure time is defined as the total time from the beginning of the mucosal incision to the completion of the lesion resection, including circumferential mucosal incision, submucosal dissection, snaring, and additional submucosal injections during each procedure.

#### Secondary outcomes

The secondary outcomes are as follows: time of the mucosal incision; time and speed of the submucosal dissection, with or without snaring; en bloc and complete resection, curability, determined by histological assessment of the resected specimen; endoscopic procedural adverse events, including intraoperative perforation, delayed perforation, and delayed bleeding; degree of the dissected submucosal layer before snaring; volume of the injection solution used; number and duration of hemostasis events using a hemostatic device during the procedure; thickness of the submucosal layer in the resected specimen; and whether there was handover to another operator.

### Subgroup analysis

A subgroup analysis will be conducted to investigate the relationship between the procedure time and tumor location, tumor size, operators’ experience, and pathological ulceration. The following four subgroups were defined: tumor location (upper or middle third of the stomach/lower third of the stomach), tumor size (0–9 mm/≥10 mm), operators’ experience with ESD (0–29 cases/≥30 cases), and pathological ulceration (negative/ positive). In the analysis of the subgroups mentioned above, ESD procedure time will be compared between H-ESD and C-ESD as a primary outcome.

### Definitions

The FAS is defined as the enrolled patients excluding those who do not receive study treatment, those with serious non-compliance with ethical guidelines, and those with missing primary endpoint data. The per-protocol set is defined as patients included in the FAS, excluding those who do not meet the inclusion criteria and those with significant deviations from the study protocol. The ESD procedure time is defined as that from the beginning of the mucosal incision to the completion of tumor resection as described above. The ESD procedure time is divided into individual procedure times, namely the procedure time for the mucosal incision and submucosal dissection with or without snaring. The incision speed is defined as the circumferential length of the resected specimen/incision time (mm/min). With or without snaring, the dissection time is defined as the time from the beginning of the submucosal dissection to the completion of the tumor resection. The dissection speed, with or without snaring, is defined as the area of the resected specimen/the time of dissection, with or without snaring (mm^2^/min). The resected specimen will be flattened on the plastic plate, and the length (mm) of its long and short axes will be measured using a ruler. These measurements will be used to calculate the circumferential length (mm) and resected area (mm^2^). En bloc resection is defined as single-piece resection. Complete resection is defined as en bloc resection with free vertical and horizontal margins. Curability is divided into A, B, C-1, or C-2 based on the Japanese gastric cancer treatment guidelines [5]. Delayed bleeding is defined as clinical evidence of bleeding after the ESD procedure requiring endoscopic hemostasis or a blood transfusion. Perforation is diagnosed when mesenteric fat or intra-abdominal space is observed with a stomach wall defect during the ESD procedure or when free air is detected on X-ray or computed tomography scans after the ESD procedure is complete. When snaring is conducted for resecting the lesion, the degree of the dissected submucosal layer by the tip of the endo-knife will be assessed. The volume of the viscous solution injected using the injection needle will also be recorded. The number of hemostasis events using hemostatic forceps that occur before resection of the lesion will be counted. The time of hemostasis is defined as the cumulative time from the appearance of the hemostatic forceps on the monitor to the completion of hemostasis. Hemostasis using the endo-knife or prophylactic hemostasis for the vessels after ESD will not be recorded. The thickness of the submucosal layer in the resected specimen will be assessed just below the center of the lesion.

We will also investigate the factors associated with ESD procedure time, including tumor morphology, location, size, pathological ulceration, and operators’ experience.

Tumor characteristics will be classified according to the Japanese classification of gastric carcinoma [20]. Tumor location will be classified as the upper third, middle third, and lower third of the stomach. Tumor position will be classified as the lesser curvature, greater curvature, anterior wall, and posterior wall. Endoscopists will be classified as either expert or non-expert based on their skill with ESD. Experts are defined as endoscopists with the experience of ≥30 ESD cases for EGN. The remaining endoscopists will be defined as non-experts.

### Randomization

Investigators from the participating institutions will have 24-h access to a Web-based central randomization system that provides immediate and concealed allocation. Eligible patients will be centrally randomized (1:1) into either the H-ESD or C-ESD group using the Web-based computer program, the Universal Hospital Medical Information Network (UMIN) Internet Data and Information System for Clinical and Epidemiological research (INDICE)-Cloud version. Randomization will be performed using dynamic balancing, which uses the minimization method by tumor location (upper third or middle third of the stomach vs. lower third of the stomach), tumor size (0–9 mm vs. ≥10 mm), and operators’ experience with ESD (0–29 cases vs. ≥30 cases). Patients and investigators will not be blinded to the allocated treatment group, and a unique patient identification number will be entered into the system to ensure anonymity.

### Blinding

Not applicable.

### Study organization

The coordinating center will be established by the principal and coordinating investigators. The former will be responsible for overseeing the trial and the latter for supporting the local investigators in trial management and data recording at each participating institution. Core members of the team will meet at least once a month or more often as required during the trial period.

The steering committee will be established by the principal investigator, coordinating investigators, statisticians, and gastroenterology experts. They will monitor and evaluate the overall conduct of the trial and make recommendations regarding trial-related decisions.

An independent data and safety monitoring board will be established by experts independent from this study and its competing interests. They will monitor the trial’s progress and confirm that it has been conducted, recorded, and reported in accordance with the trial’s protocol and relevant laws, regulations, and guidelines. Both online and on-site monitoring will be used to review the trial processes. Investigators will review data on endoscopy reports, pathological results, and adverse events up to the first discharge and enter it into the INDICE cloud. They will review and manage the input data for each case.

Participants will be free to withdraw from participation at any time upon request. An investigator may terminate participation in the study if a participant meets a newly developed or not previously recognized exclusion criterion that precludes further participation, such as the occurrence of a clinical adverse event or other medical condition or situation where continuing participation would not be in the participant’s best interest. The IRB of Kyushu University may terminate the trial in the event of a safety problem.

### Serious adverse events

The following AEs will be considered to be serious AEs (SAEs): AEs that lead to death or life-threatening AEs, AEs requiring re-hospitalization for treatment or extension of the hospitalization period, AEs leading to persistent or marked disability or dysfunction, AEs that have the potential to cause birth defects in offspring, and AEs requiring treatment to prevent the above results, even if they do not result in immediate life-threatening events, death, or hospitalization.

The reporting procedure is as follows: If investigators are aware of the occurrence of SAEs, required reporting measures will be taken by the investigators, such as providing explanations to the enrolled patients and reporting to the principal investigator in accordance with the “standard procedure manual for handling serious adverse events in medical research for humans (Kyushu University)” and in compliance with the “Ethical Guidelines for Medical and Health Research Involving Human Subjects (Japan).” If a principal investigator is aware of the occurrence of SAEs, the principal investigator will also prepare an SAE report and submit it to the hospital director. The hospital director will then submit the report to the clinical trial ethics review committee. In addition, information regarding the occurrence of SAEs will be promptly shared with all investigators. The entire study will be discontinued if the clinical trial ethics review board deems it necessary.

### Statistics

#### Hypotheses and data analysis

The primary efficacy endpoint is the procedure time of the ESD. The null hypothesis is that the procedure time for the H-ESD and C-ESD procedures in patients with EGN is the same.

Analysis of the primary and secondary outcomes will be conducted using the FAS. We plan to analyze complete cases without imputation of missing data because this trial is designed to generate little missing data.

An analysis of covariance for the logarithmically converted ESD procedure time will be conducted with tumor location (upper or middle third of the stomach vs. lower third of the stomach), tumor size (0–9 mm vs. ≥10 mm), and operators’ experience with ESD (0–29 cases vs. ≥30 cases) as covariates. H-ESD will be considered a superior treatment procedure, compared with C-ESD, if the procedure time for H-ESD is significantly shorter than that for C-ESD. A 2-sided *p*-value <0.05 will be considered to indicate statistical significance. In the sub-analysis, if a subgroup factor is one of the covariates, the analysis will be performed without including the sub-group factor as a covariate.

Statistical analyses will be performed by independent statisticians at the Data Center of the Center for Clinical and Translational Research (CCTR) at Kyushu University Hospital. The data will be transferred to SAS statistical software version 9.4 (SAS Institute INC., Cary, NC, USA) for all analyses.

#### Sample size estimation

In this study, to test the superiority of H-ESD over C-ESD, the sample size was estimated based on the primary endpoint (the logarithmically converted procedure time for ESD). According to a previous report comparing the treatment outcomes of C-ESD and H-ESD, the logarithmically converted procedure times (minutes, mean ± standard deviation (SD)) for C-ESD and H-ESD were 1.578 ± 0.225 and 1.244 ± 0.228, respectively [12]. Considering these results, we hypothesized that H-ESD would provide a 0.2 (37%) reduction in procedure time and estimated the SD of 0.3 conservatively. Based on these assumptions, a total of 74 participants is needed to reach a power of 80% for a *t*-test with a significance level of 0.05. To compensate for a 10% dropout rate, we aim to include 82 participants in the study.

#### Interim analyses

Interim analyses will not be performed. Considering the high curability and safety of H-ESD and C-ESD reported in the precious studies, patients would not be seriously disadvantaged by completing the study without an interim analysis.

### Data registration, handling, and retention

The trial database will be created by investigators entering anonymized data into the Web-based UMIN INDICE cloud. The trial data entry system and database will be secured and password-protected. The data management team and endpoint adjudication committee will be established by the principal investigator and independent statisticians at the Data Center of the CCTR at Kyushu University Hospital. Their responsibilities include the establishment of data randomization systems, electronic database report form design, data analysis, and verification. The principal investigator will retrieve the trial database after results from the last registered cases have been entered and submit it for final statistical analysis. Members of the data management team and endpoint adjudication committee will not participate in any intervention.

## Discussion

We expect that H-ESD will reduce the ESD procedure time for EGN compared with C-ESD. However, the results of two previous retrospective studies comparing the treatment outcomes of H-ESD and C-ESD are controversial. One of the reports regarding a multi-center study using a propensity score matching analysis showed a 53% reduction in procedure time for H-ESD, compared to that for C-ESD [12]. There were important limitations to this study, however, such as its retrospective nature and the use of a relatively small sample size (29 pairs after matching); furthermore, it was conducted using a propensity score matching analysis, and so there might be a confounding bias other than the factors involving matching. In contrast, another study conducted at a single center failed to demonstrate a significant difference in procedure time between H-ESD and C-ESD, although a 23% reduction was achieved using H-ESD [13]. This study was also performed retrospectively and with a small sample size (12 for C-ESD and 26 for H-ESD), both of which are important limitations. Although it was shown in this study that there were no significant differences in background characteristics of the enrolled patients between the two groups, it is not clear whether the small sample size might have affected the statistical analysis and/or the treatment outcomes. These findings further highlight the need for a multi-center RCT to confirm the benefits of H-ESD for EGN in clinical practice. Therefore, in this study, we will obtain an appropriate sample size and statistically calculate the results to further demonstrate the superiority of the H-ESD procedure time compared to that of C-ESD. Moreover, in this study, patients with EGN only who meet the “absolute indication of EMR or ESD” will be included based on the predetermined inclusion criteria, since 83% of enrolled patients met “the absolute indication” in our previous study showing the superiority of H-ESD over C-ESD [12].

The conventional EMR technique using snaring, on the other hand, has the limitation of curability. One of the disadvantages of EMR is that the en bloc resection rate is significantly lower than that of C-ESD, especially when the lesions are larger than 10 mm [8, 21, 22]. We expect that this limitation will be compensated for by H-ESD, where snaring is conducted after the circumferential incision and subsequent partial dissection. Therefore, the curability of each procedure will be set as a secondary outcome, and a sub-analysis will be performed on the data from lesions larger than 10 mm.

In the present clinical setting, lesions that fulfill the absolute indication for EMR or ESD are often performed by non-experts since ESD for these lesions is relatively easier than it is for other indicated lesions [5]. This study will, thus, include not only ESD experts but also non-experts, as operator endoscopists, to reflect real-world clinical practices, where the procedures conducted by non-experts are supervised by experts. We will then conduct a sub-analysis stratified by experts and non-experts, which will confirm its usefulness to both operators. Thus, we believe the results obtained from this study in this way will apply to clinical practice.

During the H-ESD procedure, we will use a multifunctional device (the SOUTEN high-frequency surgical knife) that will enable us to perform all procedures other than submucosal injection, including mucosal incision, submucosal dissection, and snaring, with a single device. Since performing ESD using the SOUTEN is much less expensive than it is with other conventional endo-knives, the SOUTEN may contribute to cost savings [19].

In this study, we aim to confirm the superiority of H-ESD over C-ESD for EGN. We expect that our findings will provide valuable information for determining the value of H-ESD for the standard treatment of EGN cases that fulfill the absolute indications for EMR or ESD.

## Trial status

This study was approved by the Kyushu University IRB for Clinical Trials (IRB No. 20202005) on July 28, 2020. This study was registered at UMIN-CRT (000041244) on July 29, 2020. Patients from Fukuoka or Oita prefecture in Japan are expected to participate in the trial. The trial was initiated at Kyushu University on August 1, 2020, and participation by other institutions will be allowed once IRB approval has been obtained from those institutions. Recruitment is expected to take 1 year and 8 months.

## Data Availability

Trial and data details can be obtained from the principal investigator upon reasonable request. The investigators plan to make data available through publication after completion of the study.

## Abbreviations

ER: Endoscopic resection
EGN: Early gastric neoplasms
EMR: Endoscopic mucosal resection
ESD: Endoscopic submucosal dissection
H-ESD: Hybrid ESD
C-ESD: Conventional ESD
CONSORT: Consolidated Standards of Reporting Trials
IRB: Institutional Review Board
TA-ESD: Traction-assisted ESD
RCT: Randomized controlled trial
UMIN: Universal Hospital Medical Information Network
INDICE: Internet Data and Information System for Clinical and Epidemiological research
AE: Adverse event
SAE: Severe AE
CCTR: Center for Clinical and Translational Research
